# Improved resolution of microbial diversity in deep-sea surface sediments using PacBio long-read 16S rRNA gene sequencing

**DOI:** 10.1128/msphere.00770-24

**Published:** 2024-11-12

**Authors:** Jie Gao, Ziming Wang, Wenjie Deng, Boxuan Sa, Xiaoxia Chen, Ruanhong Cai, Yi Yan, Nianzhi Jiao, Elaine Lai-Han Leung, Di Liu, Wei Yan

**Affiliations:** 1 Computational Virology Group, Etiology Research Center, Wuhan Institute of Virology, Chinese Academy of Sciences, Wuhan, China; 2 College of Marine Science and Technology, China University of Geosciences, Wuhan, China; 3 University of Chinese Academy of Sciences, Beijing, China; 4 Faculty of Health Sciences, University of Macau, Macau SAR, China; 5 State Key Laboratory of Marine Environmental Science, College of Ocean and Earth Sciences, Xiamen University, Xiamen, China; 6 Carbon Neutral Innovation Research Center, Xiamen University, Global ONCE Program, Xiamen, China; E O Lawrence Berkeley National Laboratory, Berkeley, California, USA

**Keywords:** deep-sea surface sediment, microbial diversity, 16S rRNA gene, long-read platform

## Abstract

16S rRNA gene sequencing is the gold standard for identifying microbial diversity in environmental communities. The Illumina short-read platform is widely used in marine environment studies due to its cost-effectiveness and high accuracy, but its limited read length restricts taxonomic identification mainly to genus or family levels. Recently, the PacBio long-read sequencing platform was developed. This method has exceptional base-level resolution exceeding 99%, thereby effectively mitigating the challenges associated with high error rates commonly observed in long-read sequencing technologies. However, few studies have compared the PacBio long-read and Illumina short-read platforms in marine deep-sea sediments. Here, the PacBio long-read and Illumina short-read platforms were compared with samples collected from the deep-sea surface sediments from the cold seep in the Shenhu area of the South China Sea offshore Pearl River Estuary. Comparisons revealed a more comprehensive taxonomic identification, α-diversity, and β-diversity by PacBio long-reads. The PacBio long-read platform exhibited higher classified rates and classified taxonomy at all levels, particularly at the species level. The PacBio long-read platform was also more accurate at capturing fine spatial-scale variations in microbial communities in sediments. Our studies will facilitate the selection of 16S rRNA sequencing platforms for investigating fine spatial-scale patterns in microbial communities in deep-sea surface sediments and serve as a crucial methodological reference for future studies on microbial diversity.

**IMPORTANCE:**

The PacBio long-read platform, with its exceptional base-level resolution exceeding 99%, has advanced our comprehension of deep-sea microbial diversity. By comparing microbial community analyses conducted using the Illumina short-read and PacBio long-read sequencing platforms, we have provided an enhanced understanding of fine spatial-scale patterns in microbial community diversity with depth across a deep-sea sediment core, as well as methodological insights that will be valuable for future research in this field.

## OBSERVATION

16S rRNA gene sequencing is the gold standard for identifying microbial diversity in environmental communities (1, 2). The extensive application of the Illumina short-read platform has profoundly enriched our comprehension of microbial diversity within microbial communities (3–5). Because of its cost-effectiveness and high accuracy, the Illumina short-read platform is widely applied to investigations on marine environments, such as seawater and sediments (6–9). Nonetheless, the limited read length restricts its ability to encompass merely one to two hypervariable regions of the 16S rRNA gene, which typically permits taxonomic identification only at the genus or family level (10, 11). Recently, the PacBio long-read sequencing platform, which has exceptional base-level resolution exceeding 99%, has provided enhanced precision for the analysis of microbial communities at both the species and strain levels (12).

Deep-sea surface sediments, which cover over 70% of the Earth’s surface (13, 14), harbor a huge amount of microbial biomass (15, 16). The microbes present in marine sediments are estimated to number approximately 10^29^, representing the dominant biological consortium within this environment (17, 18). Their activity in the decomposition of organic matter plays a pivotal role in driving essential biogeochemical cycles (19–21). However, only a few studies have compared the usage of the PacBio long-read and Illumina short-read sequencing platforms to investigate deep-sea sediments. Here, the PacBio long-read and Illumina short-read sequencing platforms were utilized to analyze the cold seep from the Shenhu area of the South China Sea offshore Pearl River Estuary, assessing collected deep-sea sediments. The Shenhu cold seep is known for its unique microbial community and abundant resources, which constitute a multifaceted and heterogeneous environment in the seabed with a multitude of microbial niches (22). These characteristics make deep-sea sediments from the cold seep in the Shenhu area of the South China Sea an optimal subject for testing these two sequencing techniques.

The objectives of this investigation were as follows: (i) to evaluate the effectiveness of PacBio long reads at enabling the comprehension of fine spatial-scale variations in microbial diversity within deep-sea surface sediments based on microbial composition, taxonomy rate, classified taxonomy, and alpha and beta diversities; and (ii) to assess the potential for the high-resolution sampling of deep-sea surface sediments for yielding biologically significant insights. To fulfill these objectives, we collected a deep-sea sediment core from the cold seep in the Shenhu area of the South China Sea (water depth: 1547 m; location: 115.903E, 19.8044N). We conducted a sampling reaching seabed coring depths of up to 10 cmbsf, followed by continuous vertical sampling at 1 cm intervals. A comprehensive analysis of the bacterial communities encompassing each incremental sample was performed, followed by a comparative analysis of the full-length 16S rRNA gene sequences obtained by utilizing PacBio Sequel IIe and partial 16S rRNA gene sequences with Illumina MiSeq PE250.

**Fig 1:**
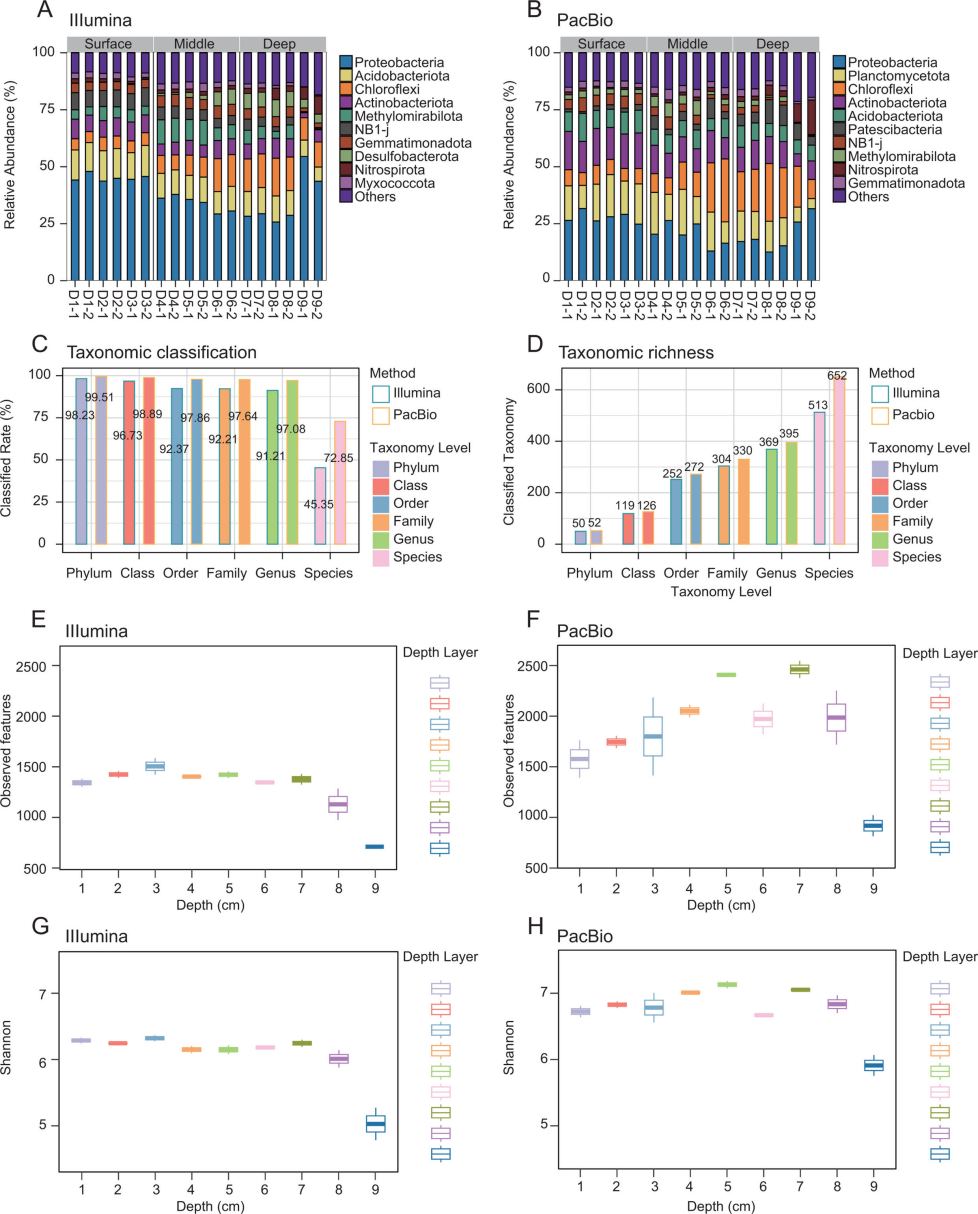
Comparative analysis of the sediment microbial communities using Illumina short-read and PacBio long-read amplicon sequencing. (A, B) Microbial composition of the sediment samples at the phylum level. The DNA from each sample from every centimeter interval was extracted and sequenced twice to generate biological replicates. (C, D) Comparison of the Illumina short-read and PacBio long-read library taxonomy classification rates of overall richness uncovered at different taxonomy levels. (E–H) Alpha diversity of the sediment samples from each sediment interval as measured by the observed ASVs and Shannon index based on the analysis using the Illumina short-read (E, G) and PacBio long-read (F, H) sequencing.

To achieve a more precise sampling, our study involved the vertical sectioning of the entire sediment core into nine distinct samples. The top section was approximately 2 cm in thickness, succeeded by the continuous extraction of the remaining eight samples at intervals of 1 cm each. The samples were stratified into three depth-related groups. The samples from 0 to 2, 2 to 3, and 3 to 4 cm were categorized as the surface layer. Samples from 4 to 5, 5 to 6, and 6 to 7 cm were classified as the middle layer, while those from 7 to 8, 8 to 9, and 9 to 10 cm represented the deep layer. Our initial analysis focused on the taxonomic annotation of the bacterial communities within these sediment samples based on Illumina short-reads and PacBio long-reads. The compositional analysis of the microbial communities revealed a more comprehensive taxonomic identification and α-diversity when based on the PacBio long reads and when compared to the Illumina short reads (Fig. 1). This enhancement was observed at all levels of classification, particularly at the species level. The PacBio long-read sequencing identified 72.85% of the OTUs (a total of 652 OTUs), while the Illumina short-read sequencing identified 45.35% of the OTUs (a total of 513 OTUs; Fig. 1C and D). These findings align with existing literature on long-read sequencing applications, demonstrating improved annotation rates for both soil and marine environments (23–25). The α-diversity of the Illumina short-read and PacBio long-read data sets was then assessed. The PacBio long-read sequencing revealed a significantly higher count of observed amplicon sequence variants (ASVs) (PacBio long reads: 2546–814 vs Illumina short reads: 1597–588) and a more elevated Shannon index (PacBio long reads: 10.36–8.30 vs Illumina short reads: 9.23–6.96), signaling a substantial increase in the measured α-diversity (Fig. 1E through H). Furthermore, distinct trends in diversity indices were noted between the two sequencing methods. In the Illumina short-read data set, the Shannon index reached a zenith before a subsequent decline with increased sediment core depth (Fig. 1G). In contrast, the PacBio long-read data set displayed an initial increase in the Shannon index, followed by a decrease as the sediment core depth progressed (Fig. 1H). The evenness index and the phylogenetic diversity (PD) are depicted in Fig. S1.

**Fig 2:**
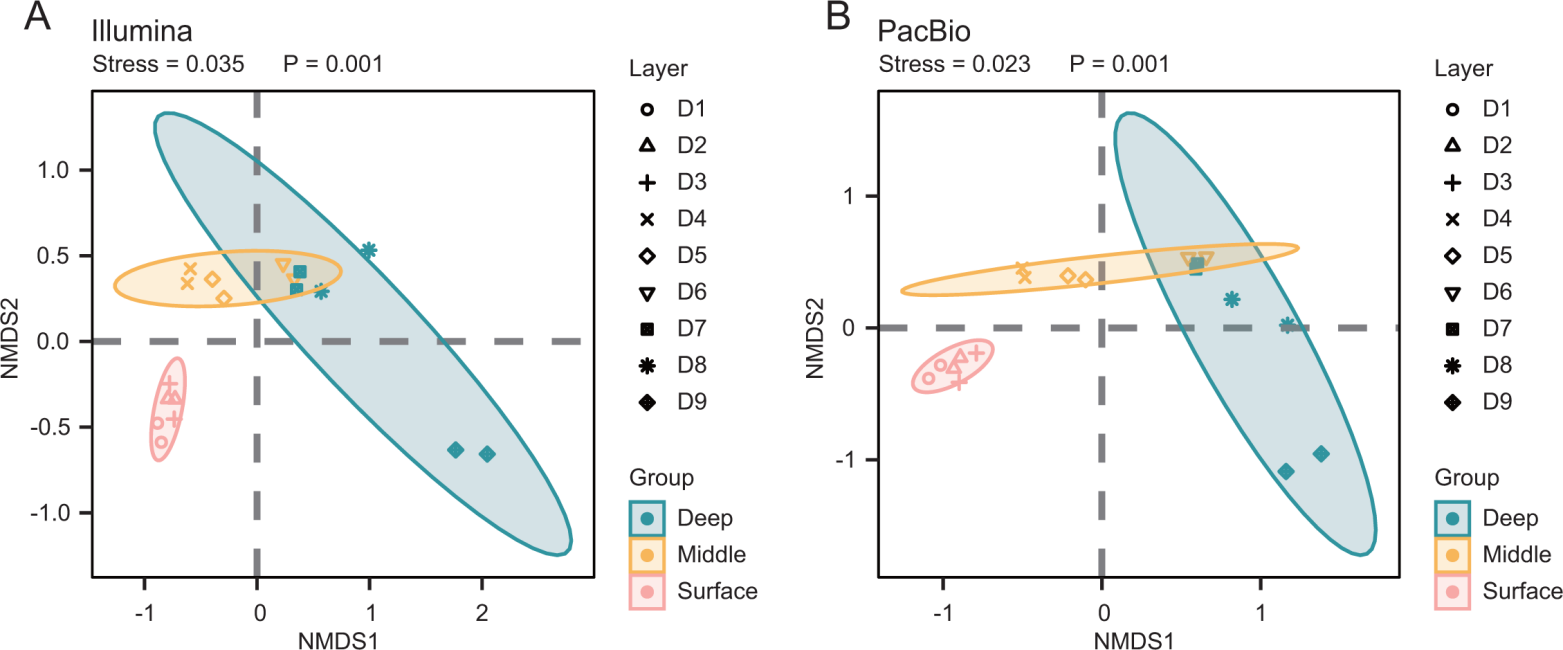
Nonmetric multidimensional scaling (NMDS). (A, B) Beta diversity of the sediment samples grouped by different depths using NMDS ordinations based on Bray–Curtis determined using Illumina short-read (A) and PacBio long-read (B) technologies.

The β-diversity analysis revealed dynamic shifts in the microbial community structure within deep-sea surface sediments that were correlated with the sediment core depth increments. Although Illumina short reads and PacBio long reads both indicated a general trend in β-diversity patterns, PacBio long reads showed more pronounced variances in the community structure (Fig. 2A and B). The non-metric multidimensional scaling (NMDS) analysis revealed a lower stress value in the PacBio long-read data set (0.023), which was markedly beneath the value obtained for the Illumina short-read data set (0.035). This indicates that PacBio long reads provide a finer differentiation of information regarding the microbial community structure. Additionally, a permutational multivariate analysis of variance (PERMANOVA) further validated the superiority of the PacBio long reads at capturing subtle details of the community structure (NGS: R^2^ = 0.339, *P* < 0.001 vs PacBio long reads: R^2^ = 0.477, *P* < 0.001). Such disparities underscore the importance of high-resolution sampling for discerning microscale patterns in deep-sea surface sediments. Studies on marine deep-sea surface sediments at reduced spatial resolutions (spanning several centimeters to tens of centimeters) have hitherto failed to encapsulate these intricate microbial community structural patterns (26–28).

The heterogeneity of the microenvironments within deep-sea surface sediments fosters a diverse array of resident microbial communities, which necessitates the selection of an appropriate sequencing methodology to elucidate the microbial diversity and the community structure of microbes present in these sediments. We employed the Illumina short reads and the PacBio long reads of the 16S rRNA gene to delineate variations in microbial communities across a 1 cm incremental gradient within the uppermost 10 cmbsf of deep-sea sediments in the cold seep. Our findings indicate that the PacBio long-read 16S rRNA gene sequencing was superior to the Illumina short-read sequencing at detecting fine spatial-scale variations in the microbial communities within these sediments. The analysis conducted using the PacBio long reads revealed more comprehensive taxonomic identification, α-diversity, and beta-diversity while enabling higher classified rates and taxonomy. These findings offered a more holistic and detailed perspective of microbial diversity. In this study, the PacBio long-read sequencing platform and its enhanced resolution significantly augmented our comprehension of the microbial diversity and community structure within deep-sea sediments; therefore, this method has the potential to provide invaluable insights into the complex microbial dynamics of these environments worldwide.

### Sample collection

The samples were obtained by the R/V “TAN KAH KEE” in the South China Sea during the summer of 2022. The sediments were collected from station Z8 (water depth: 1547 m, location: 115.903E, 19.8044N) with a box corer. From these, an approximately 10 cm-long undisturbed column was extracted using a secondary PVC tube corer. The samples were immediately sealed post-collection and stored onboard at −20°C. Upon returning to the laboratory, the core was subsampled by scraping the uncontaminated sediment from the center of the core at 1 cm intervals using sterilized spatulas. These subsamples were then frozen at −20°C for subsequent DNA extraction and physicochemical parameter analysis (29).

### DNA extraction, PCR amplification, and sequencing

The total DNA from the sediment samples was extracted using the DNeasy PowerMax Soil Kit (Qiagen, Germany). To extract DNA from each sample, two or three replicate sediment samples weighing 5 g were collected from every 1 cm increment of the core and processed. This approach was used to obtain a comprehensive DNA profile of the sediment. All steps in the DNA extraction process were performed in accordance with the manufacturer’s instructions. The DNA concentrations were quantified using a Qubit 3.0 fluorometer (Thermo Scientific).

For the Illumina short-read sequencing, the DNA samples underwent amplification of the 16S rRNA gene (V3–V4 region) using the primers 341F (5′-CCTACGGRRBGCASCAGKVRVGAAT-3′) and 806R (5′-GGACTACNVGGGTWTCTAATCC-3′). The resulting amplicons were then barcoded and sequenced on the Illumina MiSeq PE250 platform according to the manufacturer’s prescribed methods. For the PacBio long-read sequencing, a full-length 16S rRNA gene was amplified using the primers 27F (5′-AGRGTTYGATYMTGGCTCAG-3′) and 1492R (5′-RGYTACCTTGTTACGACTT-3′), after which the amplicons were sequenced on the PacBio platform according to the manufacturer’s instructions. All procedures related to PCR amplification, library preparation, and sequencing were conducted by GENEWIZ Ltd. (Suzhou, China).

### Data processing and analysis

The Illumina and PacBio amplicon data sets were processed using the open-source bioinformatics platform, QIIME2 (version 2023.7.0) (30). The “tools import” command was used to import the raw data for downstream analysis. The DADA2 plugin was used to denoise the reads and create amplicon sequence variants (ASVs) (31). For the Illumina and PacBio amplicons, the “dada2 denoise-paired” and “dada2 denoise-ccs” commands were used to infer ASVs, respectively. ASVs that appeared in only a single sample were removed. The Naive Bayes classifier mode in the feature-classifier plugin was used for taxonomic classification. The Illumina and PacBio ASVs were both assigned using a SILVA database downloaded from QIIME2 data resources (silva 138 99% OTUs full-length sequences) to infer taxonomy (32). ASVs that originated from mitochondria, chloroplast, and archaea were also removed from the data set. All libraries were subsampled against the lowest ASV number in all samples (15,000).

Alpha and beta diversities were analyzed using the R package vegan (version 2.6–4), and the similarity between samples was estimated using Bray–Curtis distances. The NMDS analysis was performed using the metaMDS function from the vegan package, and the suitability of the analysis was evaluated by examining the stress value obtained (considering ≤0.2 as reasonable). Differences among groups were tested using PERMANOVA implemented in the R package vegan. Data visualization and plotting were conducted using ggplot2.

## Data Availability

The 16S rRNA gene amplicon data pertaining to microbial communities in the surface sediments of the South China Sea, as reported herein, have been deposited in NCBI GenBank under the BioProject number PRJNA1057716.

## SUPPLEMENTAL MATERIAL

Figure S1 msphere.00770-24-s0001.pdf Comparative analysis of the sediment microbial communities using Illumina short-read and PacBio long-read amplicon sequencing.

Legends msphere.00770-24-s0002.docx Legends for the supplemental material.

Table S1 msphere.00770-24-s0003.xlsx Statistics of the Illumina short-read and PacBio long-read amplicon sequencing.

ASM does not own the copyrights to Supplemental Material that may be linked to, or accessed through, an article. The authors have granted ASM a non-exclusive, world-wide license to publish the Supplemental Material files. Please contact the corresponding author directly for reuse.
