# Supplementary figures and images for: Improved resolution of microbial diversity in deep-sea surface sediments using PacBio long-read 16S rRNA gene sequencing
Source: mSphere. 2024 Nov 12;9(12):e00770-24. doi: 10.1128/msphere.00770-24 (PMC11656776; doi:10.1128/msphere.00770-24)

Supplementary figure 1

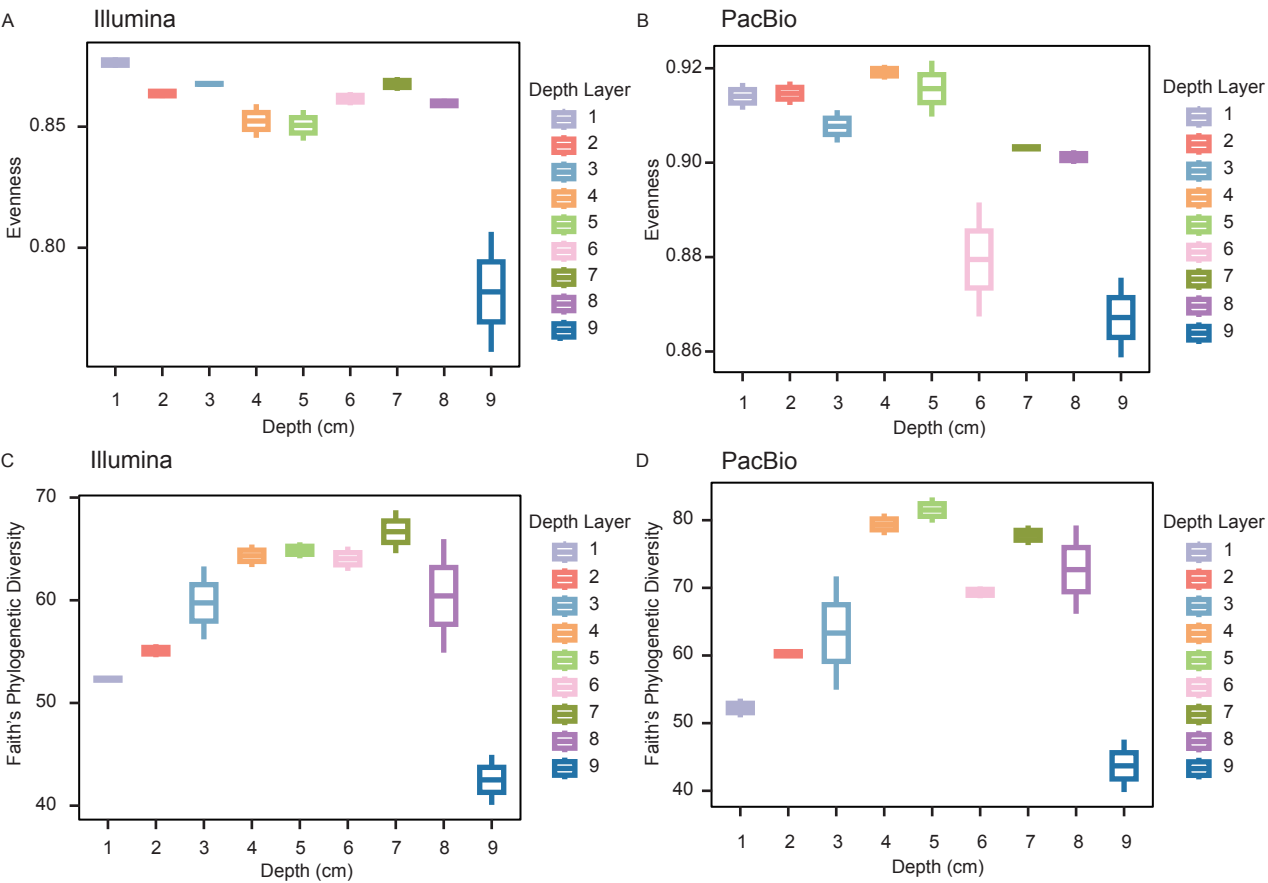

Supplement: Figure S1 — Comparative analysis of the sediment microbial communities using Illumina short-read and PacBio long-read amplicon sequencing. [file msphere.00770-24-s0001.pdf]
