# Supplementary material for: Improved resolution of microbial diversity in deep-sea surface sediments using PacBio long-read 16S rRNA gene sequencing
Source: mSphere. 2024 Nov 12;9(12):e00770-24. doi: 10.1128/msphere.00770-24 (PMC11656776; doi:10.1128/msphere.00770-24)
Supplement: Legends — for the supplemental material. [file msphere.00770-24-s0002.docx]

**Supplementary materials**

**Improved resolution of microbial diversity in deep-sea surface** **sediments using PacBio long-read 16S rRNA gene sequencing**

Jie Gao^1,2,6^, Ziming Wang^3^, Wenjie Deng^2^, Boxuan Sa^2^, Xiaoxia Chen^4,5^, Ruanhong Cai^4,5^, Yi Yan^1,6^, Nianzhi Jiao^4.5^, Elaine Lai-Han Leung^3^, Di Liu^1,6*^, Wei Yan^2,4,5*^

**AUTHOR AFFILIATIONS**

^1^Computational Virology Group, Center for Bacteria and Viruses Resources and Bioinformation, Wuhan Institute of Virology, Chinese Academy of Sciences, Wuhan, 430071, China

^2^College of Marine Science and Technology, China University of Geosciences, Wuhan, 430074, China

^3^Faculty of Health Sciences, University of Macau, Macau SAR, 999078, China

^4^State Key Laboratory of Marine Environmental Science, College of Ocean and Earth Sciences, Xiamen University, Xiamen, 361102, China

^5^Carbon Neutral Innovation Research Center, Xiamen University, Global ONCE Program, Xiamen, 361005, China

^6^University of Chinese Academy of Sciences, Beijing, 101408, China

^*^Corresponding authors.

E-mail addresses: liud@wh.iov.cn; yanwei@cug.edu.cn

**Supplementary figure 1** Comparative analysis of sediment microbial communities using Illumina short-read and PacBio long-read amplicon sequencing. (A–D)) Alpha diversity of sediment samples from each interval, as measured by Evenness and Faith’s Phylogenetic Diversity based on analysis using Illumina short-read (A, C) and PacBio long-read (B, D) technologies, respectively.

**Supplementary table 1** The statistics of Illumina short-read and PacBio long-read amplicon sequencing.
